# Impact of cognitive reserve on bipolar disorder: a systematic review

**DOI:** 10.3389/fpsyt.2023.1341991

**Published:** 2024-02-02

**Authors:** Kazuki Matsumoto, Sayo Hamatani

**Affiliations:** ^1^Division of Clinical Psychology, Kagoshima University Hospital, Research and Education Assembly Medical and Dental Sciences Area, Kagoshima University, Kagoshima, Japan; ^2^Research Center for Child Mental Development, University of Fukui, Fukui, Japan; ^3^Division of Developmental Higher Brain Functions, United Graduate School of Child Development, University of Fukui, Fukui, Japan; ^4^Department of Child and Adolescent Psychological Medicine, University of Fukui Hospital, Fukui, Japan

**Keywords:** bipolar disorder, cognitive reserve, cognition, dysfunction, systematic review

## Abstract

**Background:**

Cognitive reserve (CR) is a complex concept that includes premorbid IQ, years of education, and exposure to neuropsychological stimuli through work and leisure. Previous studies have suggested that CR has a positive impact on several aspects of bipolar disorder. Synthesizing the evidence to date is an important work in providing directions for future studies. The objectives of this systematic review to summary impact of CR on onsetting, relapsing bipolar episodes, buffering cognitive dysfunctions, and maintaining quality of life (QOL) in bipolar disorder.

**Methods:**

Two researchers independently reviewed selected paper from three database as PubMed, PsychINFO, and Web of Science. The search keywords were “bipolar disorder” and “cognitive reserve.” The selected studies were classified as the levels of evidence according to the criteria of the Oxford Center for Evidence- Based Medicine. The results of the selected studies were summarized according to the objectives.

**Results:**

Thrity six studies were included in this review. People with high CR may have fewer bipolar episodes and alleviate cognitive impairments and dysfunction. CR may keep the functional level in patients with bipolar disorder.

**Conclusion:**

The results of this systematic review suggest that CR may be involved in preventing relapse of bipolar episodes and may alleviate cognitive dysfunction. However, effect on prevention of onset-risk and relapse of bipolar episodes need further investigation in prospective studies.

**Systematic review registration:**

https://www.crd.york.ac.uk/prospero/display_record.php?ID=CRD42021270293, the protocol was registered with PROSERO (CRD42021270293).

## Background

Patients with bipolar disorder often show cognitive dysfunction even during euthymia (1, 2). The areas of cognitive dysfunction in bipolar disorder involve attention, language learning, memory, and executive function (3, 4). Seventy percent of people with bipolar disorder have some cognitive impairments, even during euthymia (5). Such differences in cognitive dysfunction among individuals may be explained by cognitive reserve (CR). CR is an essential concept for understanding cognitive health and is developed through lifelong education and curiosity of the topic. CR proxies include years of education, intelligence quotient (IQ), employment, and active social interaction during leisure. CR can be linked to degenerative brain changes associated with dementia, Parkinson’s disease, and schizophrenia (6–10).

As research progresses, the interesting facts about CR have been reported through research into cognitive functions in bipolar disorder. Relatively recent longitudinal cohort studies suggested that CR might prevent cognitive decline and alleviate dysfunction (11, 12). Cognitive impairment in bipolar disorder plays an important role in quality of life (QOL) (13). Therefore, the treatment of bipolar disorder often includes cognitive rehabilitation to improve cognitive functions (14).

However, a systematic review on previous studies found no robust evidence regarding effectiveness of cognitive rehabilitation for bipolar disorder (15). This may be the result of intervening in populations with different CR levels. Presumably under the influence of differential CR. By clarifying the impact of CR on cognitive function in people with bipolar disorder, it may be possible to find subtypes that respond to cognitive rehabilitation. We therefore conducted a systematic review to investigate the impact of CR on bipolar disorder.

The objectives of this work were to conduct systematical review investigating the influence of CR on the relapse bipolar episodes or cognitive, functional, and psychopathological manifestations in patients with bipolar disorder. The hypothesis of the present study was set as the following four: (1) High CR delay the of onset bipolar disorder; (2) high CR prolongs euthymia period; (3) high CR buffers cognitive impairment; and (4) high CR maintains QOL. Through this work, we clarify the role of CR in bipolar disorder and propose a subgroup in which cognitive rehabilitation is likely to yield positive outcomes.

## Methods

**Figure 1:**
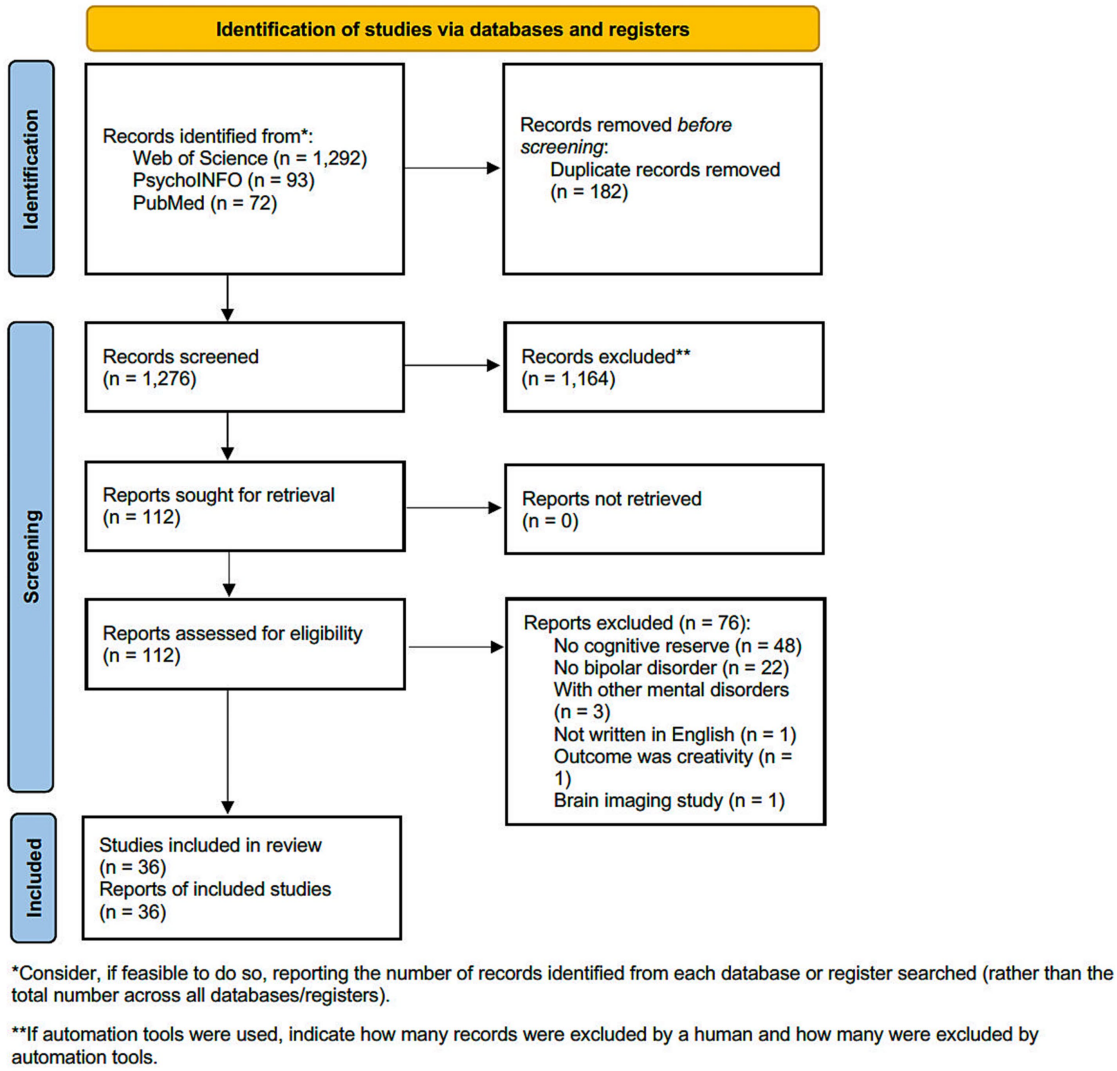
Flow diagram of this systematic review.

We conducted the present systematic review based on AMSTAR-2 (16). The current study’s protocol was registered with PROSERO (CRD42021270293). The Supplementary File contains more details of the protocol. We searched previous studies regarding CR for bipolar disorder, with the keywords “bipolar disorder” and “cognitive reserve,” using three databases: PsychINFO, PubMed, and Web of Science. Searches were last updated on December 11th, 2023. This review included all studies investigating the effects of CR proxies in bipolar disorder. We excluded studies that were based solely on neuroimaging, those that did not include CR proxies and those that included people with non-bipolar psychiatric disorders or neurological disorders. Figure 1 created according to the PRISMA guidelines, show the flowchart of the selected studies (17).

For studies that met the selection criteria and were included in the review, we rated the quality of the evidence on a five-point scale according to the criteria of the Oxford 2011 levels of evidence: level 1, systematic review of randomized controlled trials (RCT); level 2, randomized trial or observational study with dramatic effect; level 3, non-randomized or cohort study; level 4, case-series or case–control studies; and level 5, mechanism-based reasoning (18). We raised the level of evidence from two to three because appropriately designed and strictly controlled observational studies were considered valuable as experimental studies (19).

## Results

Thirty-six studies were included in the current review (11, 12, 20–53). Table 1 shows the characteristics of the studies included in the present review.

**Table 1:** Summary of the selected studies.

Author and year (Country)	Type of study and level of evidence	Sample	CR indicator	Results
Beunders et al. (2023) (42) (USA)	Longitudinal (cohort) *Level 2	963 patients with bipolar disorder aged >49 years	Years of formal education and employment status	Difference of those CR proxies did not be observed between bipolar-type I and type II.
Burdick et al. (2022) (43) (Multiple)	Longitudinal (cohort) *Level 2	5,882 patients with bipolar disorder.	Years of formal education and premorbid IQ by a reading-based task.	The low level of education was associated with functional impairment.
Cotrena et al. (2017) (21) (Brazil)	Case control *Level 4	38 patients with bipolar I disorder, 39 with bipolar II disorder, and 106 healthy controls	Reading and writing habits, IQ (the block design and vocabulary subtest from WAIS-III)	Reading and writing habits, IQ, and frequency of diagnosis predicted suppression control. IQ and diagnostic frequency predicted working memory. Cognitive flexibility was predicted by reading and writing.
Cotrena et al. (2020) (20) (Brazil)	Cross-sectional *Level 4	95 patients with bipolar disorder and 103 adults with no mood disorder	IQ (the block design and vocabulary subtest from WAIS-III) and years of formal education	People with bipolar disorder had lower executive function and functional levels than controls. IQ mediated cognitive function and affected functional levels.
Cotrena et al. (2021) (44) (Brazil)	Cross-sectional *Level 4	109 adults with bipolar disorder and 121 with no mood disorder	IQ (the block design and vocabulary subtest from WAIS-III), years of formal education, and habits of reading and writing	CR influences language fluency, cognitive flexibility, and working memory.
Correa-Ghisays et al. (2022) (45) (Spain)	Longitudinal (cohort) *Level 2	35 adults with bipolar disorder and 28 health controls	IQ (vocabulary subtest from WAIS-III) and years of formal education	Adults with bipolar disorder show lower CR compared to healthy adults, while there is no difference in general intelligence.
Donaldson et al. (2003) (22) (England)	Case control *Level 4	43 patients with bipolar I disorder	Full scale IQ (WAIS-III)	Low IQ in childhood may not be associated with the development of bipolar type I disorder.
Drakopoulos et al. (2020) (23) (Sweden)	Longitudinal (cohort) *Level 2	120 patients with bipolar disorder	Full scale IQ (WAIS-III) and occupational functioning (work of studying)	Univariate analyses revealed better overall cognitive function in active patients in terms of IQ and executive functioning.
Eyler et al. (2022) (46) (Multiple)	Cross-sectional *Level 4	805 patients with both manic and depressive symptoms	Years of formal education and employment status	Only years of formal education showed a difference between the asymptomatic group and the other group. The asymptomatic group had a relatively higher level of education.
Forcada et al. (2015) (24) (Spain)	Cross-sectional *Level 4	52 patients with bipolar disorder and 49 healthy controls	Education-occupation, leisure activities, and premorbid IQ	Euthymic patients with bipolar disorder had lower executive function, verbal, and visual memory performance than healthy controls. CR predicted cognitive and functional outcomes.
Forlenza et al. (2022) (47) (Multiple)	Cross-sectional *Level 4	Individuals aged 50 and above with bipolar disorder who have obtained information about lithium treatment.	Years of formal education and employment status	There was no difference in CR between the group with favorable prognosis under lithium treatment and the group with unfavorable prognosis under lithium treatment
Fuentes et al. (2016) (25) (Mexico)	Cross-sectional *Level 4	34 patients with bipolar disorder (12 with low compliance to the treatment and 22 with high compliance)	Years of formal education	Patients with low level of compliance performed significantly worse than those with high treatment compliance on various memory tests. Years of education was an important factor for executive function and processing speed.
Gale et al. (2013) (26) (Sweden)	Cross-sectional *Level 4	60 patients with bipolar disorder and 60 healthy controls	Verbal IQ (a reading test) and non-verbal IQ (a progressive matrices test)	Despite comparable IQ levels, patients with bipolar disorder completed fewer years of education than controls.
Garcia-Laredo et al. (2015) (27) (Spain)	Cross-sectional *Level 4	24 patients with bipolar disorder and 15 healthy controls	Years of education	There was no difference between the groups in terms of verbal fluency performance. Years of education impacted semantic verbal fluency and correlated with phonemic verbal fluency.
Gilbert et al. (2010) (28) (USA)	Longitudinal (cohort, 15–43 months of follow-up) *Level 2	154 patients with bipolar I disorder	Years of education	Baseline concentration problems and years of education significantly predicted employment.
Glahn et al. (2006) (29) (USA)	Longitudinal (cohort, 22.6 years of follow-up) *Level 2	1,049,607 Swedish man.	IQ was measured at a mean age of 18.3 years using four written subtests of verbal, logical, spatial, and technical ability.	Compared to men with average verbal ability, risk of hospitalization with pure bipolar disorder rose as verbal ability increased, such that men with the highest verbal ability had a 41% increase in risk; men with the lowest verbal ability had an increase in risk of 34%.
Hinrichs et al. (2017) (30) (UAS)	Longitudinal (cohort, 5 years of follow-up) *Level 2	1,059 patients with bipolar disorder and 54 healthy controls	CR (verbal IQ and years of education)	The overall relative rate of change in cognitive function did not differ between the bipolar disorder and healthy control groups. Intellectual ability may be a protective factor against cognitive decline.
Jimenez et al. (2017) (31) (Spain)	Cross-sectional *Level 4	113 patients with bipolar disorder	The estimated IQ (the vocabulary subtest from the WAIS-III)	A higher estimated IQ may act as a protective factor against cognitive decline in this group of patients.
Koenen et al. (2009) (32) (UK)	Longitudinal (cohort, 32 years of follow-up) *Level 2	1,037 healthy children	IQ (WISC-R)	Higher childhood IQ predicted increased risk of adult mania.
Lima et al. (2019) (34) (Brazil)	Case–control *Level 4	Of bipolar disorder, 30 with intact cognition, 23 with selective cognitive impairment, and 16 with global cognitive impairment	The estimated IQ (the vocabulary and matrix reasoning subtest from WAIS-III) and Years of education	The intact group had more years of education and a higher estimated IQ than globally and selectively impaired subgroups of patients with bipolar disorder.
Lin et al. (2020) (35) (China)	Cross-sectional *Level 4	125 bipolar I disorder and 60 healthy controls	Premorbid intelligence (the vocabulary subtest from the WAIS-R) and educational level (total years of education completed)	Premorbid intelligence significantly moderated the associations between the number of bipolar episodes and neurocognitive functioning, and the educational level also moderated the relationships between the total number of bipolar episodes and subjective cognitive functioning.
Levy et al. (2011) (33) (USA)	Longitudinal (cohort, 3 months of follow-up) *Level 3	82 patients with bipolar disorder	The estimated IQ (WAIS vocabulary and block design subtest), years of education and employment state	Significant group differences emerged on the measure of IQ. There was no difference in years of education and employment between those with bipolar disorder, whose remission was maintained, and those who relapsed at 3 months of follow-up.
Martino et al. (2008) (36) (Argentina)	Cross-sectional *Level 4	50 patients with bipolar disorder and 30 healthy controls	Premorbid IQ (the WAIS vocabulary subset)	Patients with cognitive functioning within normal limits had higher psychosocial functioning and premorbid IQ.
Martino et al. (2017) (37) (Argentina)	Cross-sectional *Level 4	119 patients with bipolar disorder and 40 healthy controls	Premorbid IQ (the WAIS vocabulary subset)	Premorbid IQ might moderate the relationship between the number of hypo/manic episodes and executive function.
Posoni et al. (2020) (38) (Brazil)	Cross-sectional *Level 4	111 patients with bipolar disorder and 91 patients with major depressive disorder	CR scores calculated by principal component analysis of the estimated IQ (the vocabulary and block design subtests in WAIS-III), years of education, and frequency of reading and writing	CR may be protective against cognitive impairment in patients with bipolar disorder.
Tabarés-Seisdedos et al. (2008) (11) (Spain)	Longitudinal (cohort, one-year of follow-up) *Level 3	43 patients with bipolar disorder, 25 healthy controls	The estimated premorbid IQ (the vocabulary subtest of the WAIS-R) and years of education	Executive function, attention, and working memory were lower in those with bipolar disorder than in healthy subjects at the time of 1 year follow-up. Years of education and estimated IQ did not explain occupational adaptation, but executive function and reasoning ability did.
Tsapekos et al. (2020) (39) (UK)	Cross-sectional *Level 4	80 patients with bipolar disorder: 25 cognitively intact; 15 selective deficits in verbal learning and memory; 30 intermediate deficits across all cognitive domains; 10 severe deficits across all domains	CR estimated three proxy measures: years of education and two premorbid intelligence scales (the vocabulary subtest in WAIS-III and the test of premorbid function)	Cognitive functioning decline after the onset of bipolar symptoms was most pronounced in severe cases, with the lowest cognitive reserve.
Sato et al. (2023) (48) (Japan)	Cross-sectional *Level 4	24 individuals with bipolar disorder and 24 healthy adults.	Educational years, pre-illness IQ (vocabulary), and leisure activity history were examined.	CR was found to be correlated with cognitive functions related to language fluency and memory.
Solé et al. (2022) (49) (Spain)	Longitudinal (cohort, 2 years follow-up) *Level 2	92 individuals with bipolar disorder	Educational level (years), Occupation (not working), Estimated IQ (vocabulary subtest, processing speed, working memory in WAIS-III)	Language memory and inhibitory control explained a more favorable functional outcome over the long term.
de Sales et al. (2023) (50) (Brazil)	Cross-sectional *Level 4	89 individuals with bipolar disorder and 69 healthy controls.	The vocabulary subtest of the Wechsler Abbreviated Scale for Intelligence and the Hopkins Verbal Learning Test – Revised	Individuals with bipolar disorder perform lower on theory of mind’s tasks compared to healthy controls. Vocabulary, a CR proxy, is not correlated with theory of mind task performance in individuals with bipolar disorder.
Strassning et al. (2018) (12) (USA)	Longitudinal (cohort, 20 years of follow-up) *Level 2	87 patients with bipolar disorder: 46 employed; 41 non-employed	Occupation (interview of gainful employment)	Patients with bipolar disorder who had full-time employment had a negative correlation with depressive symptoms. Employment was positively correlated with cognitive test performance.
Tsapekos et al. (2021) (51) (UK)	Cross-sectional *Level 4	80 euthymic participants with bipolar disorder	Educational level (years), premorbid IQ (the test of premorbid function)	Regarding clinically significant cognitive impairment, factors identified include lower premorbid IQ, older age, a higher number of prescribed psychotropic medications, fewer received psychotherapy sessions, and specific current psychotropic medication use.
Ringin et al. (2023) (52) (Multiple)	Longitudinal (cohort, 5 years of follow-up) *Level 2	1,074 individuals with bipolar disorder aged 40–70 and 59,653 healthy controls.	Employment status, university/college degree, and leisure activities (physical activity, habits of exposure to intellectual stimuli while sitting)	There were no differences in education level and employment status between individuals with bipolar disorder and healthy controls. Physical activity was slightly higher in individuals with bipolar disorder. Those who spent longer hours using a computer for non-work-related activities had relatively higher cognitive function, regardless of whether they had bipolar disorder. Among individuals with bipolar disorder, those with high levels of physical activity had lower cognitive function than those with low activity levels.
Romero et al. (2016) (40) (Argentina)	Cross-sectional *Level 4	46 patients with bipolar II disorder and 46 healthy controls	The premorbid IQ (vocabulary subtest in WAIS-III)	Significant correlations were identified in bipolar patients regarding hyperthymic temperament, verbal memory, and premorbid IQ.
Ryan et al. (2013) (41) (USA)	Cross-sectional *Level 4	156 patients with bipolar disorder and 143 healthy controls	Occupation using the DIGS and the premorbid IQ (vocabulary subtest in WAIS-III)	Patients with bipolar disorder who were unemployed/unable to work exhibited greater difficulties processing emotional information and on executive function as compared to those who were employed, independent of other factors. The premorbid IQ may not be relevant to getting a responsible job.
Xie et al. (2023) (53) (China)	Cross-sectional *Level 4	64 patients with bipolar disorder and 58 healthy controls	The Cognitive Reserve Assessment Scale in Health (CRASH)	CR is related to functional outcomes.

Table 1 the selected studies CR in patients with bipolar disorder has been suggested to be partially lower than in healthy controll. Eyler et al. (46) analyzed data from 805 patients with bipolar disorder across 12 international studies. They investigated symptom severity in asymptomatic control groups, those predominantly experiencing depressive symptoms, predominantly manic patients, and those with bipolar mood symptoms. Exploratory analyses also considered demographic data such as education level, employment status, age, onset age, and gender. Among these, only education level showed a difference between the asymptomatic group and the other groups (46). In a study that constructed CR with fewer proxies, individuals with bipolar disorder appeared to have lower CR than the healthy population. Correa-Ghisays et al. (45) compared CR in 35 individuals with bipolar disorder to 28 healthy controls. Cognitive reserve was calculated using the Vocabulary subtest of WAIS-III and formal years of education. The bipolar group demonstrated lower cognitive reserve compared to healthy adults, although there was no difference in general intelligence (IQ) (45).

In CR proxies, this review summarizes that high premorbid IQ was associated with the onset of bipolar disorder. Low intelligence in childhood was not identified as a risk factor for bipolar disorder; rather, a 32-year birth cohort study suggested that high childhood IQ increased the risk of bipolar disorder in middle age (22, 32). A study by Gale et al. (26) showed that men who developed diagnostic bipolar disorder were more intelligent than the general population. Results of a previous study demonstrate that it is unknown whether educational background influences the development of bipolar disorder (29). Glahn et al. (29) reported that patients with bipolar disorder had less years of education than healthy controls, suggesting that the onset of the disease impaired academic achievement. Of IQ-adjusted bipolar disorder and healthy control students, more than 60% enrolled in college, with 47% of the healthy control group and 16% of the bipolar disorder group earning a college degree. However, when comparing the degree of educational achievement of respondents with bipolar disorder after the age of 25 years (*n* = 16) with the healthy control group, there was no significant difference in educational level.

For the number of bipolar episodes, it has been suggested that it may be related to premorbid IQ, however, the results of this review are inconsistent. While two previous studies claimed that a higher premorbid IQ was associated with a higher frequency of bipolar episodes (34, 40), another two studies provided opposite results (33, 37). A previous study that provides the highest level of evidence of these, is the study conducted by Levy et al. (33). A three-month follow-up study of post-discharge bipolar individuals conducted by Levy et al. (33) found that patients who were not re-hospitalized had a significantly higher estimated premorbid IQ. Examining the estimated IQ components of this study, readmission patients were significantly worse with block design, but no such difference was seen in the vocabulary. In other words, among the components of premorbid IQ, poor spatial grasping ability and nonverbal analogical ability may predict the risk of readmission. Furthermore, there is evidence that stable employment contributes to fewer bipolar episodes. Patients with higher levels of education have been reported to experience fewer bipolar episodes (34). Xie et al. (53) utilized The Cognitive Reserve Assessment Scale in Health (CRASH) as an assessment tool to measure cognitive reserve, consisting of IQ, education/occupation, and leisure activities. CRASH is implemented through a semi-structured interview and is considered adaptable for clinical use. Individuals with bipolar disorder exhibited slower functional outcomes, premorbid IQ, working memory, language fluency, and processing speed compared to healthy adults. Among individuals with bipolar disorder, those with higher CRASH scores were correlated with longer intervals between current episodes. In other words, ther results of this study suggested that higher cognitive reserve may potentially delay the recurrence of bipolar episodes (53).

For severity of CR and mood problems, Strassnig et al. (12) examined CR based on estimated IQ using WAIS-III subtests of vocabulary and block design, years of education, and frequency of reading and writing in a longitudinal study of 20 years. The results of this study suggested that full-time employment was negatively correlated with depressive symptoms (12). Forlenza et al. (47) compared the demographic and clinical characteristics of elderly individuals with bipolar disorder receiving lithium treatment to those not using lithium. Unsurprisingly, the group undergoing lithium treatment exhibited clinically better prognosis and higher functional levels. The analysis data in this study included CR proxies such as educational history and occupational status. Regarding education and occupational status, there were no differences between the lithium-treated group with a favorable prognosis and the non-lithium-treated group with an unfavorable prognosis. However, factors related to more severe bipolar disorder, such as family history of mental disorders, presence of rapid cycling, and severe depressive states, were evident between the groups (47). Beunders et al. (42) conducted a cohort study on individuals with bipolar disorder aged 50 and above, comprising 963 cases. The study did not find any relationship between educational history and employment status with bipolar disorder subtypes. However, type-I individuals had a higher frequency of hospitalization compared to type-II. While hospitalization is considered an indicator of more severe symptoms, this study can not assert that CR influences bipolar disorder subtypes or severity differences, as educational history and employment status were roughly comparable between the groups. The relationship between CR and clinical symptoms within each group was not investigated in this study.

Martino et al. (36) reported that the proportion of respondents with cognitive impairment was 62, and 38% were intact: premorbid IQ appears to be higher in patients with intact cognitive function. Other studies have similarly suggested that high premorbid IQ reduces cognitive impairment (23, 30, 34). A five-year cohort study by Hinrichs et al. (30) found that premorbid IQ was correlated with change of performance for cognitive functional tasks pertaining to visuospatial ability, verbal memory, and simple visual motor attention, in patients with various states of bipolar disorder. Three studies have reported that educational level may also protect against declining cognitive functions (23, 27, 31). Cotrena et al. (44) conducted a study involving 109 individuals with bipolar disorder. They used educational history and estimated IQ derived from the Block Design and Vocabulary subtests of WAIS-III, as well as reading and writing habits, as CR proxies. The study suggested CR in bipolar disorder influences working memory, language fluency, and cognitive flexibility (44).

Employment opportunities (in at least three studies) have also been reported to protect cognitive function (11, 23, 41). Drakopoulos et al. (23) suggested that respondents with active work opportunities had a better overall cognitive function than those who were inactive. Tabarés-Seisdedos et al. (11) investigated the relationship between employment opportunities and overall disability levels as measured by the total score of the 12-item WHO disability Assessment Schedule 2.0 (WHODAS-12) through a one-year cohort study. WHODAS-12 assesses six areas, namely: understanding and communication, self-care, mobility, interpersonal relationships, work and family roles, and community participation (54). This study shows that overall dysfunction can be predicted through baseline employment status (11). Ryan et al. (41) suggested that work opportunities may affect emotional processing, language fluency, and protection of processing speed. Burdic et al. (43) investigated data from 5,882 individuals with bipolar disorder (BP) who participated in 13 cohort studies conducted in seven countries. Functional impairment was present in 41–75% of patients, and it was associated with lower education levels, a higher frequency of previous mood episodes, the presence of comorbid substance use disorders, and a higher total number of psychotropic medications. Additionally, lower community functioning was related to depressive symptoms (43). In Solé et al. (49), 92 BP patients participated in a longitudinal study, with 62 completing the two-year observation period, measuring education level, occupation, estimated IQ, and cognitive function. The regression model did not detect the influence of cognitive remediation (CR) proxies, showing that only language memory and inhibitory control explained a more favorable long-term functional outcome (49).

Ringin et al. (52) conducted a cohort study in the UK from 2005 to 2010, targeting 502,649 participants aged 40–69. The study investigated correlations between cognitive function and employment status, the presence of a university/college degree, physical activity levels, and habits of exposure to intellectual stimuli while sitting. There were no differences in education level and employment status between the bipolar disorder group and the healthy control group. However, individuals with BP had slightly higher levels of physical activity. Interestingly, individuals who used computers for non-work-related activities, regardless of the presence of BP, exhibited poorer cognitive abilities compared to those who used computers less frequently. Individuals with BP who engaged in high levels of physical activity tended to have lower cognitive scores. This trend was more pronounced in individuals with exceptionally high levels of physical activity, where cognitive performance was even worse. The results of this study, analyzing the relationship between cognitive tasks and multiple CR proxies, are particularly intriguing. In summary, engaging in leisure activities that require complexity and attention may lead to higher cognitive function in the future, while high levels of physical activity may not only protect but potentially worsen cognitive function (52). Tsapekos et al. (51) conducted a study aimed at identifying predictors of cognitive impairment in 80 euthymic participants with bipolar disorder. The study examined sociodemographic and clinical factors related to cognitive performance. The results, obtained through logistic regression, identified predictors of clinically significant cognitive impairment, including lower IQ, older age, a higher number of prescribed psychotropic medications, fewer received psychotherapy sessions, and specific current antipsychotic medication use (51).

In the study by de Sales et al. (50), the premobid IQ estimated through the Vocabulary subtest of WAIS-III and the Hopkins Verbal Learning Test – Revised (HVLT-R) did not show a significant correlation with performance on theory of mind tasks. Theory of mind involves the acquisition of the ability to understand others’ perspectives, explaining the foundation of a person’s sociality (55). This research suggests that, despite slightly lower performance on theory of mind tasks, cognitive reserve does not allow for a discussion about social cognitive function. Gilbert et al. (28) showed that levels of education impacted semantic verbal fluency and correlated with phonemic verbal fluency. Sato et al. (48) compared cognitive remediation (CR) between 24 patients with bipolar disorder and 24 healthy adults. This study compared estimated IQ, years of education, and the amount of leisure activities measured using a simple test assessing the ability to correctly read Japanese ‘KANJI’ characters (JART) between the bipolar disorder group and the healthy control group. Within the bipolar disorder patient group, multiple regression analysis demonstrated an influence of estimated IQ and the amount of leisure activities on the performance of the Japanese Verbal Learning Test (JVLT) (*p* = 0.01) (48). However, since JART essentially measures vocabulary, conducting regression analysis between JART and JVLT, which also measures vocabulary, may not be appropriate: both variables are essentially measuring the same test.

Three constructs that utilize the CR index in a complex manner are for example: years of education, premorbid IQ, and leisure activities. Furthermore, studies have investigated the relationship between CR, cognition, and function in patients with bipolar disorder. Forcada et al. (24) reported that CR consisting of years of education, premorbid IQ, and leisure activities could significantly predict functionality level, executive function, and visual memory in a linear regression model analysis. Based on the results, the authors concluded that CR was an important predictor of cognitive and psychosocial function in outpatients with bipolar disorder. Martino et al. (37) also found that patients with bipolar disorder with high complex CR performed better in the following multiple cognitive functions: attention, language fluency, language memory, and learning ability, than patients without bipolar disorder (37). Ponsoni et al. (38) investigated the effects of CR and diagnosis on the measurement of working memory, inhibition, attention, language fluency, and cognitive flexibility. However, when including patients with major depressive disorder, CR had a significant effect on all cognitive variables, with the high CR group consistently outperforming the low CR group.

An important finding regarding the potential for improving CR function was reported by Cotrena et al. (21). The results of this study suggested that daily cognitive stimulation by reading/writing may make a significant positive contribution to executive function areas, such as: working memory, inhibitory control, and cognitive flexibility in patients with bipolar disorder, even in the absence of continuing education. The authors concluded that these and other forms of routine cognitive stimuli should be further emphasized in bipolar disorder intervention programs (21). In this regard, Cotrena et al. (20), by using path analysis, suggested that CR and age were mediated by cognitive ability in executive function, and indirectly affected the function of patients with bipolar disorder.

## Discussion

This systematic review summarized the current evidence of CR in bipolar disorder to suggest a subgroup in which cognitive rehabilitation is likely to yield positive outcomes. The systematic review included thrity-six studies: Twenty were cross-sectional studies (20, 24–27, 31, 35–41, 44, 46–48, 50, 51, 53), thirteen were longitudinal cohort studies (11, 12, 23, 28–30, 33, 42, 43, 45, 49, 52), and three were case–control studies (21, 22, 34). This review suggests that high IQ increases the risk of developing bipolar disorder, while high IQ is associated with fewer bipolar episodes and protects against cognitive impairment. High levels of education and stable employment also appear to protect against cognitive impairment. In contrast, if patients with bipolar disorder have low premorbid IQ, low levels of education, and an insecure employment status, they are more likely to have a poor prognosis−high frequency of future bipolar episodes and/or cognitive functional decline. Patients with bipolar disorder with these characteristics are the ones who need cognitive rehabilitation. Since exposure to cognitive stimulation has positive effects on several cognitive functions (21), high-risk populations of bipolar disorder with the features we identified may benefit.

The results of this review could not confirm the evidence that adult bipolar disorder can be prevented even with high IQ. Gale et al. (26) found a 22.6-year cohort study of 1,049,607 Swedish men that had a U-shaped relationship between premorbid IQ and the development of bipolar disorder. Verbal IQ appeared to be a good predictor of the development of bipolar disorder. In this study, 41% of respondents with high language proficiency and 34% with low language proficiency were at an increased risk of being hospitalized for bipolar disorder compared to those with average language proficiency. This result explained the increased risk of developing bipolar disorder in individuals with both low and high premorbid intelligence, which was found in previous studies. Low educational history may predict the onset of the disorder (29). However, the previous studies included in the present review assessed few CR proxies, such as premorbid IQ and educational history. Future studies should investigate the role of comprehensive CR proxies, such as employment and leisure.

High IQ, educational level and stable employment appear to be predictive factors for the low frequency of bipolar episodes. Bipolar disorder is a recurrent disorder, and early awareness and treatment of prodromes is central to recurrence prevention strategies (56). High CR may contribute to this process. It was suggested that employment opportunities had the tendency to reduce the frequency of depressive episodes, even if the condition had deteriorated due to bipolar disorder (11, 41). Extremely low activity levels were often associated with depressive symptoms (57), and social participation, such as social gatherings with friends and neighbors, often contributed to mood enhancement (58). Depressive symptoms also reduced social participation by reducing motivation and general interest (59). This review suggested that in bipolar disorder, the presence or absence of occupational opportunities included in CR proxies prolonged remission and promoted a good prognosis. Physical activity at work may regulate the social rhythm, which represented the core of treatment in interpersonal and social rhythm therapy, which was shown to be effective in treating bipolar disorder (60–63). Having a profession and working daily assisted in regulating strong social rhythms. Therefore, it was natural that CR proxies, such as professions and leisure activities, were associated with the regulation of social rhythm. A study comprising 1,501 adult women suggested that physical activity reduced the risk of depression, whereas occupational activities had no effect in this regard (64). Therefore, future studies should include a sub-analysis of occupations with active and inactive physical activity when examining employment opportunities as CR proxies.

From the perspective of social resources, it is possible for people with higher IQs and higher levels of education to deal with the onset of a bipolar episode, because they tend to have a higher occupational status and rich resources (65–68). Populations comprising individuals with high intelligence and education levels tend to be of high social status, where cognitive rehabilitation may be less effective. Individuals with bipolar disorder who have a worse prognosis may, in contrast, belong to the handicapped population. Therefore, in the future, the effects of cognitive rehabilitation should be investigated in prospective randomized controlled trials that consider social status.

Several studies also provided evidence that high CR reduced hospitalizations (25, 33). There was evidence that education level was related to high therapeutic compliance (25), which also demonstrated that CR was an important factor in long-term mood stability. However, a study by Fuentes et al. (25) found that there was no difference in bipolar episodes. Recognizing prodromes of bipolar episodes can encourage adaptive behavior for the prevention of bipolar episodes (69–71). Individuals with high levels of education and high IQ could have an advantage in this process (46). The insights currently submitted through the review are mostly based on studies examining correlations, making it challenging to infer causal relationships. As is well-known, when treatment adherence is high, mood stability is maintained, and cognitive function is preserved in patients with bipolar disorder (72). In bipolar disorder, insufficient treatment adherence is often observed, leading not only to compromised treatment effectiveness and poor outcomes but also significantly increasing the risk of relapse, rehospitalization, and suicide (73–75). Establishing treatment alliance between physicians and patients is believed to contribute to treatment adherence, and it is suggested that patients with high cognitive reserve—meaning sufficient intellectual function and interpersonal skills—may find it easier to build such alliances. Moreover, there is evidence that psychological education regarding medication therapy and daily lifestyle for individuals with bipolar disorder can yield favorable outcomes (76). In summary, having knowledge about bipolar disorder and its treatment likely contributes to their favorable prognosis. Both the high cognitive reserve (CR) and treatment compliance enhance opportunities for acquiring knowledge about the disorder and its treatment. Therefore, these aspects are considered crucial perspectives in supporting patients with bipolar disorder. Further research is needed to elucidate the impact of these factors on prognosis. Specifically, conducting prospective randomized cohort studies is essential to verify whether patients with high CR indeed exhibit better outcomes.

We also found that patients with low education levels were more likely to have cognitive decline (31, 43). Premorbid IQ in most of the studies included in this review was estimated from the WAIS-III vocabulary. Since education increased linguistic fluency (27), verbal IQ may be more likely to predict cognitive-functional outcomes than the IQ of CR proxies. Education was associated with stable employment status (28), and long-term dysfunction may be alleviated if employment opportunities are available (41). People with bipolar disorder could have improved cognition when exposed to daily cognitive stimuli after the onset of bipolar disorder (21). In addition, Cotrena et al. (20) found that CR mediated executive function and indirectly affected patient function. Exposure to cognitive stimuli, such as reading and writing, helps to maintain cognitive functions after onset, because the degree of executive dysfunction was a major factor in the prognosis of patients with bipolar disorder (23, 77). Furthermore, individuals with bipolar deiroser exposed to intellectual stimuli through computer-related activities during leisure showed a tendency to have higher cognitive function (52). Our findings were consistent with cognitive rehabilitation and psychotherapy, which increased the possibility of experiencing intellectual stimulation (78). CR could improve cognitive function in individuals with bipolar disorder, and indirectly increase their functional level (79, 80). Several RCTs focusing on cognitive remediation therapy aimed at enhancing metacognitive functions have shown improvement in neurocognitive mechanisms in bipolar disorder (14, 81). When interpreting this evidence collectively, it seems natural to conclude that activities generating intellectual stimuli, such as engagement in work during occupation or intellectual hobbies, as reflected by some cognitive remediation proxies, have a positive impact.

This systematic review had three limitations. First, most studies evaluated CR in a reactive (rather than proactive) manner. Bipolar disorder is known to involve subthreshold depression even during remission. Depressive symptoms affect IQ; thus, studies should be conducted in the future to assess the risk of developing bipolar disorder based on pre-symptomatic IQ. Second, there were problems in comparing and integrating research results because various methods were used to estimate CR. Additionally, some studies calculated complex CR indexes. An international agreement between researchers on how to assess CR may be needed to consider comprehensive CR indicators and the impact of each CR proxy. The third limitation was that most of the studies included in this review had cross-sectional designs. Cross-sectional studies cannot distinguish whether CR acts upon the risk of developing bipolar disorder and the cognitive as well as functional prognosis or is simply a psychosocial variable related to the improvement of cognitive and functional performance. In the future, the effectiveness of interventions that include strictly controlled longitudinal cohort studies, educational experiences, and leisure activities should be validated in prospective randomized controlled trials. Last, since early onset of bipolar disorder is associated with a greater familial risk and unfavorable clinical outcomes (82), future research should involve studies that adjust for patient background variables such as age and gender.

## Conclusion

The objectives of this study were to response the following three hypothesis: (1) High CR delay the of onset bipolar disorder; (2) high CR prolongs euthymia period; (3) high CR buffers cognitive impairment; and (4) high CR maintains QOL. The evidence found in our systematic review suggested that high levels of education and stable employment also seem to protect against cognitive impairment. The respondents with bipolar disorder, who have low premorbid IQ, low levels of education, and insecure employment status, may be a population most in need of cognitive rehabilitation. To our knowledge, this study is the first systematic review regarding the effects of CR on the onset of bipolar disorder, relapse of bipolar episodes, and outcomes of cognition and dysfunction.

## Data availability statement

The raw data supporting the conclusions of this article will be made available by the authors, without undue reservation.

## Author contributions

KM: Writing – original draft, Conceptualization, Data curation, Formal analysis, Methodology, Project administration, Resources, Software, Validation, Visualization. SH: Funding acquisition, Writing – review & editing, Data curation, Formal analysis, Methodology, Resources, Software, Supervision, Visualization.
