# Supplementary material for: Impact of cognitive reserve on bipolar disorder: a systematic review
Source: Front Psychiatry. 2024 Feb 2;14:1341991. doi: 10.3389/fpsyt.2023.1341991 (PMC10869457; doi:10.3389/fpsyt.2023.1341991)
Supplement: Supplementary file 1 [file Data_Sheet_1.PDF]

NHS
National Institute for Health Research

UNIVERSITY of York
Centre for Reviews and Dissemination

Systematic review

A list of fields that can be edited in an update can be found here

1. * Review title.

Give the title of the review in English

Cognitive reserve of bipolar disorder: a systematic review

2. [1 change] Original language title.

For reviews in languages other than English, give the title in the original language. This will be displayed with the English language title.

3. * Anticipated or actual start date.

Give the date the systematic review started or is expected to start.

02/08/2021

4. * [1 change] Anticipated completion date.

Give the date by which the review is expected to be completed.

25/10/2021

5. * [2 changes] Stage of review at time of this submission.

This field uses answers to initial screening questions. It cannot be edited until after registration.

Tick the boxes to show which review tasks have been started and which have been completed.

Update this field each time any amendments are made to a published record.

The review has not yet started: No

Review stage	Started	Completed
Preliminary searches	Yes	Yes
Piloting of the study selection process	Yes	Yes
Formal screening of search results against eligibility criteria	Yes	Yes
Data extraction	Yes	Yes
Risk of bias (quality) assessment	Yes	Yes
Data analysis	Yes	Yes

Provide any other relevant information about the stage of the review here.

6. * Named contact.

The named contact is the guarantor for the accuracy of the information in the register record. This may be any member of the review team.

Kazuki Matsumoto

Email salutation (e.g. "Dr Smith" or "Joanne") for correspondence:

Dr Matsumoto

7. * Named contact email. [1 change]

Give the electronic email address of the named contact.

axpa0219@staff.kanazawa-u.ac.jp

8. Named contact address [1 change]

Give the full institutional/organisational postal address for the named contact.

Kakuma, Kanazawa-City, Ishikawa, Japan

9. Named contact phone number. [1 change]

Give the telephone number for the named contact, including international dialling code.

81 08 0762645988

10. * Organisational affiliation of the review. [1 change]

Full title of the organisational affiliations for this review and website address if available. This field may be completed as 'None' if the review is not affiliated to any organisation.

Laboratory of neuropsychology, Institute of Liberal Arts and Science, Kanazawa University

Organisation web address:

https://neuropsychol.w3.kanazawa-u.ac.jp/en/home-en/

11. * Review team members and their organisational affiliations. [2 changes]

Give the personal details and the organisational affiliations of each member of the review team. Affiliation refers to groups or organisations to which review team members belong. **NOTE: email and country now MUST be entered for each person, unless you are amending a published record.**

Assistant/Associate Professor Kazuki Matsumoto. Division of Clinical Psychology, Kaogshima University Hospital
Assistant/Associate Professor Sayo Hamatani. Research Center for Child Mental Development, University of Fukui

12. * Funding sources/sponsors.

Details of the individuals, organizations, groups, companies or other legal entities who have funded or sponsored the review.

This work was supported by the Japan Society for the Promotion of Science KAKENHI Grant-in-Aid for

Lifelong sciences: Reconceptualization of development of aging in the super aging society.

Grant number(s)

State the funder, grant or award number and the date of award

20H05800

13. * Conflicts of interest.

List actual or perceived conflicts of interest (financial or academic).

None

14. Collaborators.

Give the name and affiliation of any individuals or organisations who are working on the review but who are not listed as review team members. **NOTE: email and country must be completed for each person, unless you are amending a published record.**

15. * Review question.

State the review question(s) clearly and precisely. It may be appropriate to break very broad questions down into a series of related more specific questions. Questions may be framed or refined using PI(E)COS or similar where relevant.

The objective of this work is to conduct a systematic review investigating the influence of cognitive reserve (CR) on the relapse bipolar episodes or cognitive, functional, and psychopathological manifestations in patients with BP. The hypothesis of the present study are set as the following three: (1) low CR increases the risk of developing bipolar disorder; (2) high CR prolongs remission period; (3) high CR buffers cognitive impairment and keeps the functional level.

16. * Searches.

State the sources that will be searched (e.g. Medline). Give the search dates, and any restrictions (e.g. language or publication date). Do NOT enter the full search strategy (it may be provided as a link or attachment below.)

We will conduct cross-search with the keywords "bipolar disorder" and "cognitive reserve" using used three databases: PsycINFO, PubMed, Web of Science. This review will include previous articles written in English published by June 17, 2021.

17. URL to search strategy.

Upload a file with your search strategy, or an example of a search strategy for a specific database, (including the keywords) in pdf or word format. In doing so you are consenting to the file being made publicly accessible. Or provide a URL or link to the strategy. Do NOT provide links to your search **results**.

Alternatively, upload your search strategy to CRD in pdf format. Please note that by doing so you are consenting to the file being made publicly accessible.

Yes I give permission for this file to be made publicly available

18. * Condition or domain being studied.

Give a short description of the disease, condition or healthcare domain being studied in your systematic review.

Bipolar disorder is a mental disorder that causes dramatic shifts in a person’s mood, energy and ability to think clearly. People with bipolar experience high and low moods—known as mania and depression—which differ from the typical ups-and-downs most people experience. The average age-of-onset is about 25, but it can occur in the teens, or more uncommonly, in childhood. The condition affects men and women equally, with about 2.8% of the U.S. population diagnosed with bipolar disorder and nearly 83% of cases classified as severe.

19. * Participants/population.

Specify the participants or populations being studied in the review. The preferred format includes details of

both inclusion and exclusion criteria.

We designate participants in a pediatric cohort investigating the development of bipolar disorder, adolescents and adults with bipolar disorder, as the target population.

20. * Intervention(s), exposure(s).

Give full and clear descriptions or definitions of the interventions or the exposures to be reviewed. The preferred format includes details of both inclusion and exclusion criteria.

Our systematic review aims to investigate the effects of cognitive reserves. Cognitive reserve is a psychosocial construct that includes premorbid IQ, years of education, employment opportunities, and novel cognitive stimuli in leisure. Therefore, exposure of this review includes the following cognitive reserve proxies: education, employment, leisure social involvement and learning.

21. * Comparator(s)/control.

Where relevant, give details of the alternatives against which the intervention/exposure will be compared (e.g. another intervention or a non-exposed control group). The preferred format includes details of both inclusion and exclusion criteria.

For the objective of this study, we will adopt previous studies compared bipolar disorder patients with high and low cognitive reserves.

22. * Types of study to be included.

Give details of the study designs (e.g. RCT) that are eligible for inclusion in the review. The preferred format includes both inclusion and exclusion criteria. If there are no restrictions on the types of study, this should be stated.

We will include all studies investigating the effects of cognitive reserve proxies for patients with bipolar disorder.

23. Context.

Give summary details of the setting or other relevant characteristics, which help define the inclusion or exclusion criteria.

24. * Main outcome(s).

Give the pre-specified main (most important) outcomes of the review, including details of how the outcome is defined and measured and when these measurement are made, if these are part of the review inclusion criteria.

Cognitive reserve as the main outcome is measured by premorbid IQ, years of education, employment opportunities, background information on leisure activities and several tests. For example, premorbid IQ is most often estimated by Wechsler Adult Intelligence Scale 3rd edition (WAIS-III) vocabulary and block design tasks.

Measures of effect

Please specify the effect measure(s) for you main outcome(s) e.g. relative risks, odds ratios, risk difference, and/or 'number needed to treat.

25. * Additional outcome(s).

List the pre-specified additional outcomes of the review, with a similar level of detail to that required for main outcomes. Where there are no additional outcomes please state 'None' or 'Not applicable' as appropriate to the review

Additional outcomes are several clinical/functional measures: For example, Hamilton Depression Scale (HAM-D), Young Mania Rating Scale (YMRS), Trail Making Test, and Wisconsin Card Sorting Test, etc.

Measures of effect

Please specify the effect measure(s) for you additional outcome(s) e.g. relative risks, odds ratios, risk difference, and/or 'number needed to treat.

26. * Data extraction (selection and coding).

Describe how studies will be selected for inclusion. State what data will be extracted or obtained. State how this will be done and recorded.

We will adopt previous studies investigated bipolar episode recurrence, clinical symptoms, and functional levels in the relationship between CR and bipolar disorder. We will exclude studies based on brain imaging, studies that did not include cognitive reserve proxy, and studies that included people with non-bipolar disorder, psychiatric disorders or neurological disorders.

27. * Risk of bias (quality) assessment.

State which characteristics of the studies will be assessed and/or any formal risk of bias/quality assessment tools that will be used.

We will rate the quality of the evidence on a five-point scale according to the criteria of the Oxford 2011 levels of evidence (OCEBM Levels of Evidence Working Group, 2011).

[1 change] 28. * Strategy for data synthesis.

Describe the methods you plan to use to synthesise data. This **must not be generic text** but should be **specific to your review** and describe how the proposed approach will be applied to your data. If meta-analysis is planned, describe the models to be used, methods to explore statistical heterogeneity, and software package to be used.

Because cognitive reserve is a neuropsychological construct, not an intervention, to conduct meta-analysis is not suitable as a method of synthesis. Hence, we adopt narrative synthesis. We will perform narrative synthesis of each aspect to assess the effect of cognitive reserve for onset of bipolar disorder, the relapse of bipolar episodes, and cognitive and dysfunctional prognosis. In particular, we plan to describe the educational history, premorbid IQ, and knowledge of employment opportunities, which are cognitive reserve proxies, for each of the above three themes, respectively. The criteria for the data to be integrated is a study

that includes findings on bipolar disorder and the cognitive reserve proxy described above, and that all studies have a longitudinal (cohort) or cross-sectional study design. The outcomes included in the integrated data are the age of onset of bipolar disorder, the number of bipolar episodes, the severity of clinical symptoms, and the results of functional psychological tests. For outcomes regard with clinical symptom and functional test, typically, will be included Young Manic Rating Scale (YMRS), Hamilton Depression Rating Scale (HAMD), Wisconsin Card Sorting Test (WCST), Stroop test, etc.

29. * Analysis of subgroups or subsets.

State any planned investigation of ‘subgroups’. Be clear and specific about which type of study or participant will be included in each group or covariate investigated. State the planned analytic approach.

No subgroup analysis is planned at this time.

30. * Type and method of review.

Select the type of review, review method and health area from the lists below.

Type of review

Cost effectiveness

No

Diagnostic

Yes

Epidemiologic

No

Individual patient data (IPD) meta-analysis

No

Intervention

No

Living systematic review

No

Meta-analysis

No

Methodology

No

Narrative synthesis

No

Network meta-analysis

No

Pre-clinical

No

Prevention

Yes

Prognostic

Yes

Prospective meta-analysis (PMA)

No

Review of reviews

No

Service delivery

No

Synthesis of qualitative studies

No

Systematic review

Yes

Other

No

Health area of the review

Alcohol/substance misuse/abuse

No

Blood and immune system

No

Cancer

No

Cardiovascular

No

Care of the elderly

No

Child health

No

Complementary therapies

No

COVID-19

No

Crime and justice

No

Dental

No

Digestive system

No

Ear, nose and throat

No

Education

No

Endocrine and metabolic disorders

No

Eye disorders

No

General interest

No

Genetics

No

Health inequalities/health equity

No

Infections and infestations

No

International development

No

Mental health and behavioural conditions

Yes

Musculoskeletal

No

Neurological

No

Nursing

No

Obstetrics and gynaecology

No

Oral health

No

Palliative care

No

Perioperative care

No

Physiotherapy

No

Pregnancy and childbirth

No

Public health (including social determinants of health)

No

Rehabilitation

No

Respiratory disorders

No

Service delivery

No

Skin disorders

No

Social care

No

Surgery

No

Tropical Medicine

No

Urological

No

Wounds, injuries and accidents

No

Violence and abuse

No

31. Language.

Select each language individually to add it to the list below, use the bin icon to remove any added in error.

English

There is not an English language summary

32. * Country.

Select the country in which the review is being carried out. For multi-national collaborations select all the countries involved.

Japan

33. Other registration details.

Name any other organisation where the systematic review title or protocol is registered (e.g. Campbell, or The Joanna Briggs Institute) together with any unique identification number assigned by them. If extracted data will be stored and made available through a repository such as the Systematic Review Data Repository (SRDR), details and a link should be included here. If none, leave blank.

34. Reference and/or URL for published protocol.

If the protocol for this review is published provide details (authors, title and journal details, preferably in Vancouver format)

Add web link to the published protocol.

Or, upload your published protocol here in pdf format. Note that the upload will be publicly accessible.

Yes I give permission for this file to be made publicly available

Please note that the information required in the PROSPERO registration form must be completed in full even if access to a protocol is given.

35. Dissemination plans.

Do you intend to publish the review on completion?

Yes

Give brief details of plans for communicating review findings.?

36. Keywords.

[1 change]

Give words or phrases that best describe the review. Separate keywords with a semicolon or new line. Keywords help PROSPERO users find your review (keywords do not appear in the public record but are included in searches). Be as specific and precise as possible. Avoid acronyms and abbreviations unless these are in wide use.

Bipolar disorder; Bipolar episode; Cognitive reserve; Cognitive impairment; Dysfunction; Systematic review.

37. Details of any existing review of the same topic by the same authors.

If you are registering an update of an existing review give details of the earlier versions and include a full bibliographic reference, if available.

38. * Current review status. [1 change]

Update review status when the review is completed and when it is published.New registrations must be ongoing so this field is not editable for initial submission.

Please provide anticipated publication date

Review_Completed_not_published

39. Any additional information.

Provide any other information relevant to the registration of this review.

40. Details of final report/publication(s) or preprints if available.

Leave empty until publication details are available OR you have a link to a preprint (NOTE: this field is not editable for initial submission). List authors, title and journal details preferably in Vancouver format.

Give the link to the published review or preprint.
